# What’s that noise—tackling sound pollution in the NICU: a systematic review

**DOI:** 10.3389/fped.2026.1798036

**Published:** 2026-06-12

**Authors:** Vito Giordano, Sophie Mandl, Lisa Bartha-Doering, Christoph Reuter, Matthias Bertsch, Brigitte Wildner, Sophie Stummer, Katrin Klebermass-Schrehof, Marcus Maeder, Angelika Berger, Philipp Deindl

**Affiliations:** 1 Department of Pediatrics and Adolescent Medicine, Division of Neonatology, Pediatric Intensive Care and Neuropediatrics, Comprehensive Center for Pediatrics, Medical University of Vienna, Vienna, Austria; 2 Department of Pediatrics and Adolescent Medicine, Division of Pediatric Pulmonology, Allergology and Endocrinology, Medical University of Vienna, Vienna, Austria; 3 SInES (Space for Interdisciplinary Experiments on Sound), University of Vienna, Vienna, Austria; 4 Department of Music Physiology, University of Music and Performing Arts Vienna, Vienna, Austria; 5 University Library, Medical University of Vienna, Vienna, Austria; 6 Chair of Vibroacoustics of Vehicles and Machines, Technical University of Munich, Munich, Germany; 7 Department of Neonatology and Pediatric Intensive Care Medicine, University Children’s Hospital, University Medical Center Hamburg-Eppendorf, Hamburg, Germany; 8 Department of Pediatrics and Adolescent Medicine, Hospital Lüneburg, Lüneburg, Germany

**Keywords:** neonatal intensive care unit (NICU), noise level, environmental noise, preterm infants, sound exposure, review

## Abstract

**Background:**

Preterm infants are highly susceptible to environmental influences due to the immaturity of their sensory and neurological systems. In neonatal intensive care units (NICUs), ambient noise frequently exceeds recommended thresholds and may interfere with auditory and neurodevelopmental maturation. During a critical period of sensory refinement, infants are exposed to unpredictable, high-intensity soundscapes generated by alarms, respiratory devices, and staff activity, contrasting sharply with the regulated acoustic environment of the womb.

**Aim:**

This systematic review aimed to identify, describe, and categorize noise sources in the NICU and to examine their potential impact on preterm infants.

**Method:**

A systematic search across six major databases [MEDLINE, Embase, APA Psychinfo, CINHAL, Scopus, Web of Science (WOS); 2000–2026] identified studies reporting 24-h noise levels, day–night variation, and differences between incubator-internal and external sound. Eligible studies were synthesized and visualized using forest, strip, and dot plots, interpreted relative to American Academy of Pediatrics (AAP) recommendations.

**Result:**

From 113 included studies, most reported sound levels substantially above recommended limits. Alarms, staff conversations, and incubator-associated equipment were consistently identified as major contributors. Respiratory support devices produced the highest internal noise levels, at times exceeding overall NICU ambient noise. Interventions such as staff training, behavioral adjustments, and environmental modifications yielded short-term improvements but rarely produced sustained reductions.

**Conclusion:**

NICU noise is a modifiable risk factor with significant developmental implications. Future strategies should integrate environmental design, technological innovation, and infant-focused interventions at the incubator–device interface to create safer, developmentally supportive acoustic conditions. Evidence also suggests that excessive shielding may reduce meaningful auditory input, raising concerns about potential auditory deprivation.

## Introduction

The World Health Organization (WHO) estimates that more than 10 million preterm infants are born each year worldwide, and advances in neonatal intensive care have significantly improved survival rates in this population (1–3). Despite these improvements, preterm birth remains a major risk factor for impaired brain maturation, neurodevelopmental delays, and sensory impairments, particularly among extremely preterm infants (4–11). In addition to medical fragility, these infants are exposed to a highly technological environment that differs substantially from the conditions experienced in the womb. Among the various environmental stressors present in neonatal intensive care units (NICUs), environmental noise has emerged as an important concern for neonatal health and developmental care (12–17).

During the perinatal period, the auditory system undergoes rapid and critical development. Structural formation of the auditory apparatus begins around 15 weeks of gestation, while functional maturation occurs between approximately 23 and 29 weeks of gestation (18). By around 24 weeks of gestational age, preterm infants are capable of responding to auditory stimuli, highlighting the importance of appropriate auditory input for the maturation of both auditory and neurological pathways (19–21). However, because extremely preterm infants are born during this sensitive developmental window, they may be exposed prematurely to environmental sounds that differ markedly from the physiological auditory conditions of the intrauterine environment (22).

The intrauterine acoustic environment provides a naturally filtered and relatively stable soundscape. Maternal tissues, uterine walls, and amniotic fluid attenuate high-frequency external sounds while transmitting predominantly low-frequency and rhythmic signals such as maternal heartbeat, breathing, and vascular flow. As a result, fetal auditory exposure is generally characterized by continuous, low-frequency sounds rarely exceeding approximately 30 dB (20, 21, 23). In contrast, the NICU environment exposes preterm infants to a complex and often unpredictable acoustic landscape dominated by intermittent high-frequency sounds generated by medical equipment, monitoring alarms, ventilation systems, and staff activity.

For this reason, several organizations have proposed noise thresholds for hospital and neonatal care environments (14, 24–28) (Table 1). The American Academy of Pediatrics (AAP) recommends maintaining sound levels below 45 dB during the daytime and 35 dB during the night, with the aim of minimizing physiological stress, promoting sleep stability, and protecting the developing auditory system (20, 28). These recommendations are broadly aligned with international hospital acoustic guidelines proposed by the WHO, which suggest environmental noise levels of approximately 35–40 dB in patient care areas (14, 29).

**Table 1:** Recommended noise levels for hospital and neonatal care environments. Adapted with permission from the journal Building and Environment, published by Elsevier: Do neonates hear what we measure? Assessing neonatal ward soundscapes at the neonates' ears, by Bhan Lam et al., 2025; License number: 6250180977249.

Organization	Environment	Recommended noise level	Notes	Source
American Academy of Pediatrics (AAP)	NICU (day time)	≤45 dB	Recommended average sound level to reduce physiological stress and protect auditory development in preterm infants	AAP Committee on Environmental Health
American Academy of Pediatrics (AAP)	NICU (night time)	≤35 dB	Lower nighttime levels recommended to promote sleep stability and neurological development	AAP Committee on Environmental Health
American Academy of Pediatrics (AAP)	Peak sound levels	≤65 dB	Maximum transient sound exposure recommended	AAP Committee on Environmental Health
World Health Organization (WHO)	Hospital patient rooms	≤35 dB	Recommended background sound level in hospital care areas	WHO Environmental Noise Guidelines
World Health Organization (WHO)	Night-time hospital environment	≤30 dB	Recommended to protect sleep and physiological recovery	WHO Environmental Noise Guidelines
International Electrotechnical Commission (IEC)	Infant incubator (internal noise—normal use)	≤55–60 dB(A)	Maximum sound level inside incubator; updated versions suggest Leq,1 h ≤ 55 dB(A) and peak limits	IEC 60601-2-19:2020
Department of Health (UK)—HTM 08-01	Hospital wards (daytime)	≤45 dB LAeq	Recommended indoor ambient noise level for patient areas	HTM 08-01: Acoustics (2013)
Department of Health (UK)—HTM 08-01	Hospital wards (night-time)	≤35 dB LAeq	Lower limits to support patient rest and recovery	HTM 08-01: Acoustics (2013)
Department of Health (UK)—HTM 08-01	Maximum noise levels (night)	≤55 dB LAmax	Limits for intermittent noise events during night	HTM 08-01: Acoustics (2013)
Standards Australia/Standards New Zealand	Hospital wards (general)	40–45 dB LAeq	Recommended design sound levels for patient care areas	AS/NZS 2107:2016
Standards Australia/Standards New Zealand	Patient rooms (night)	30–35 dB LAeq	Lower range recommended for sleeping/resting conditions	AS/NZS 2107:2016
Standards Australia/Standards New Zealand	Intensive care/critical care areas	∼40–45 dB LAeq	Slightly higher allowance due to equipment and monitoring needs	AS/NZS 2107:2016
Consensus Committee (2020)	NICU (continuous background noise)	≤45 dB(A) Leq	Recommended hourly equivalent continuous sound level in NICU	Recommended Standards for Newborn ICU Design, 9th ed.
Consensus Committee (2020)	NICU (hourly L10)	≤50 dB(A) L10	Sound level exceeded 10% of the time	Recommended Standards for Newborn ICU Design, 9th ed.
Consensus Committee (2020)	NICU (peak levels)	≤65 dB(A) Lmax	Maximum transient sound exposure	Recommended Standards for Newborn ICU Design, 9th ed.

Adapted from Lam et al. (100); License number: 6250180977249.

Nevertheless, numerous studies have shown that noise levels in NICUs frequently exceed these limits due to the cumulative effects of medical equipment, alarms, staff activity, and architectural characteristics of the unit (30).

Exposure to excessive noise in the NICU has been associated with both acute physiological responses and potential longer-term developmental concerns. Acute responses described in the literature include alterations in heart rate, fluctuations in oxygen saturation, increased stress responses, and disturbances in sleep–wake cycles (31–35). These immediate physiological effects reflect the limited capacity of preterm infants to regulate environmental stimuli and maintain autonomic stability. Sustained exposure to high or unpredictable sound levels has also been associated with broader physiological consequences, including changes in autonomic, metabolic, and endocrine function (35, 36). At the same time, increasing attention has been directed toward the possible long-term neurodevelopmental implications of early noise exposure, although the current evidence remains heterogeneous and largely observational (37, 38). Recent investigations continue to highlight that environmental noise levels in NICU settings may frequently exceed recommended thresholds and vary depending on clinical activity and spatial organization of the unit (37).

Although incubators provide partial attenuation of environmental noise, they do not fully protect infants from acoustic exposure (17, 39, 40). In fact, incubators can also contain internal noise sources, including respiratory support devices, alarms, and airflow systems. These internal sound sources contribute to a complex acoustic microenvironment surrounding the infant that remains insufficiently characterized (17). High-frequency sounds generated within incubators may further contribute to physiological stress and interfere with neurological development, emphasizing the importance of understanding noise exposure both in the broader NICU environment and within the incubator itself (15, 16).

Taken together, these observations suggest that excessive noise should be recognized as a modifiable environmental factor with potential implications for physiological stability, auditory development, and long-term neurodevelopment in preterm infants. A clearer understanding of the characteristics and sources of NICU noise is therefore essential to inform clinical practice and environmental design strategies aimed at improving the sensory environment of hospitalized newborns.

### Aim

The present review aims to explore noise level within NICU environments and describe their acoustic characteristics. By synthesizing the available evidence to date, this review seeks to improve understanding of the NICU auditory environment and its potential implications for preterm infants. Increasing awareness of NICU noise exposure may help inform clinical practice, guide environmental design strategies, and support the development of interventions aimed at creating quieter and more developmentally supportive care environments for this highly vulnerable population.

## Methods

### Eligibility criteria

Studies were considered eligible if they reported quantitative measurements of noise levels within NICU environments, which constituted the primary outcome of this review. The focus on objective noise measurements allowed for a consistent assessment of sound exposure levels within NICU settings.

During the manuscript selection process, papers containing relevant information but not focusing on the primary outcome, were maintained and data extracted accordingly but considered as secondary outcomes. Based on the available data, the following were considered secondary outcomes: (i) day–night variation in noise levels, (ii) sound levels measured around/within the incubators (iii) the reported efficacy of interventions aimed at reducing noise exposure.

Studies were excluded if they were conference papers, abstracts, publications written in languages other than English, or if extraction of relevant quantitative information was not possible.

### Data extraction

This systematic review of the literature aimed to descriptively synthesize and report published data on noise levels within the NICU. The search strategy was developed together with the University Library of the Medical University of Vienna. Specifically, an Information Specialist (BW) screened the following databases: MEDLINE, Embase, APA Psychinfo, CINHAL, Scopus, Web of Science (WOS), using the following keyword combination: “Neonatal Intensive Care Unit”, “NICU”, “Incubator” “Intensive Care Unit”, “ICU”, “Newborn”, “Baby”, “Preterm”, “Noise”, “Level”, “Exposure”, “Intensity”, “Acoustic”, “Sound”, “Pollution”, “Decibel”. A more precise description of our search strategy can be found in the Supplementary Material (Supplement file ST1).

The search strategy included studies published between 2000 and April 14, March 2026. Three authors (VG, SM, LBD) independently extracted the relevant data. For each study, acoustic and contextual variables were systematically extracted and organized into a standardized dataset. The following parameters were collected: authors, year of publication, region/country, NICU level, type of incubator, type of ventilators, ventilation mode, mean NICU noise level, operational noise ranges, operational mean, noise levels during day vs. night, noise sources surrounding or located within the incubator, and noise-reduction interventions and their effects on both NICU environment and infant outcomes. Daytime, nighttime, and 24-h mean sound levels (dB) were reported only when explicitly provided in the original study. When studies reported sound levels as any type of ranges, these were recorded under “Operational Levels,” and an approximate central value was calculated as the arithmetic mean of the minimum and maximum values and reported as “Operational Mean.” Peak levels were extracted when explicitly available.

Where studies reported multiple independent measurement conditions (e.g., different rooms, NICU sections, or exposure scenarios), each condition was treated as a separate observation and labeled systematically (e.g., Author [A], Author [B], etc.), preserving within-study variability. In some cases, however, given the complexity and variability of reported information, data were aggregated in mean values.

For interventional studies, the type of intervention was recorded (e.g., behavioral), and pre- and post-intervention sound levels were extracted only when directly measured and explicitly reported.

Risk of bias was determined using an adapted version of the “*JBI Critical Appraisal Checklist for analytical cross-sectional studies*” (Supplementary Table S1), and refers exclusively to the noise measurement relative to the primary outcome of this study.

### Analysis & data presentation

**Figure 1:**
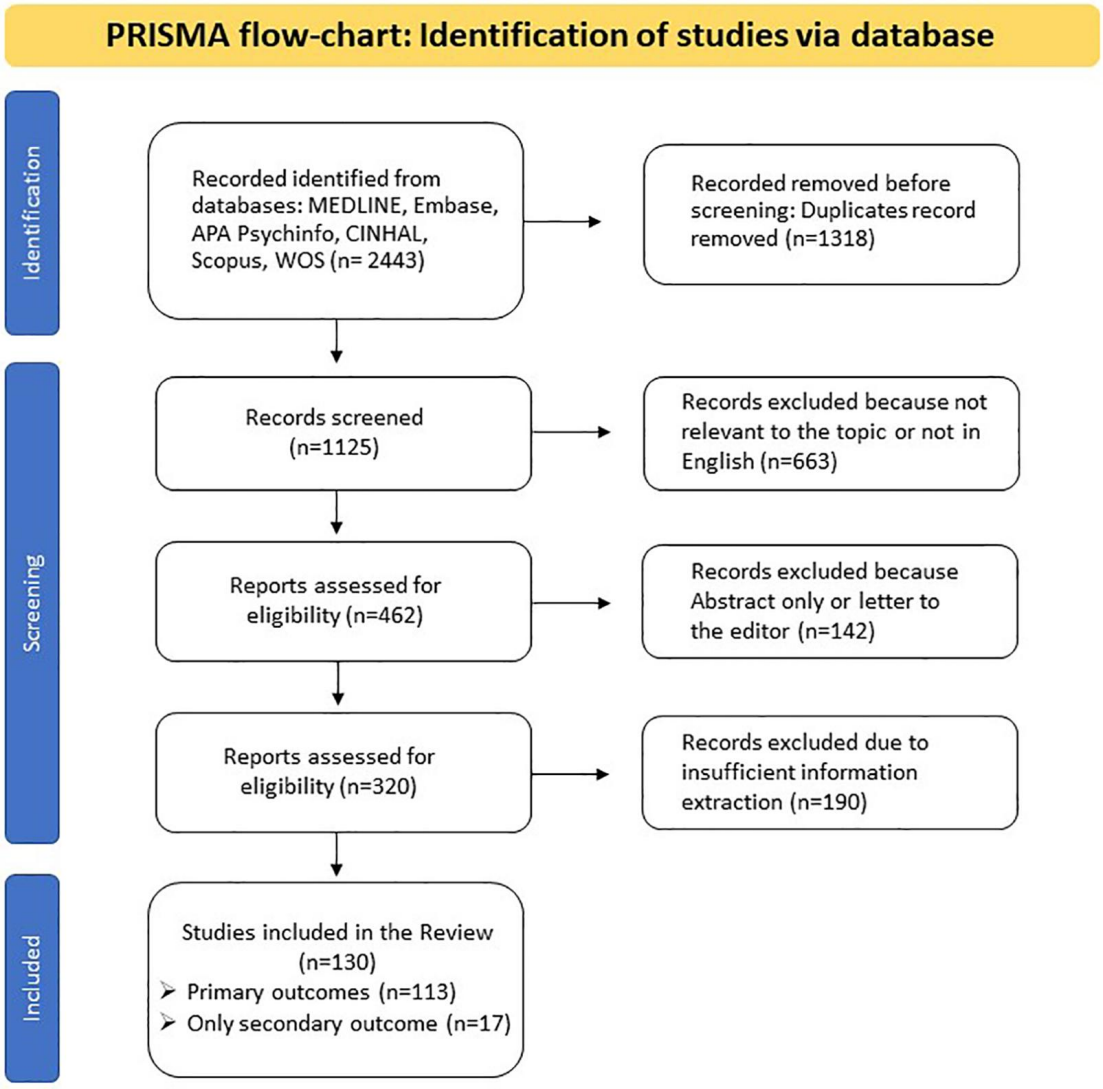
PRISMA diagram representing database screening and selection flow-chart.

Decibel values were visualized using forest plots, contextualized with reference thresholds defined by the American Academy of Pediatrics (AAP). Analysis was performed using R Studio (R Foundation for Statistical Computing, Vienna, Austria); Excel® (Microsoft Office 16 version); and IBM SPSS Statistics® (IBM Corporation, Armonk, NY). Continuous variables are reported as mean ± standard deviation (SD), while categorical variables are presented as frequencies and percentages. The study selection process followed PRISMA guidelines and is illustrated in a dedicated flow diagram (Figure 1). Geographical distribution of the included studies was visualized using a choropleth map combined with proportional symbol mapping. To examine variation in noise levels across the day, we used a strip plot (categorical *y*-axis, continuous *x*-axis) for 24-h data, and a dot plot to illustrate day-night differences. Noise sources were grouped into 4 categories (staff talking, alarms, incubator motor, incubator handling) in order to provide detailed and structured description of noise dynamics in the NICU environment.

## Results

The initial database search identified 2,443 articles. After removing duplicates, excluding irrelevant titles, and screening for full-text availability, 113 studies were deemed relevant to the primary aim of this review (20, 41–152). The selection process is illustrated in the PRISMA flow diagram (Figure 1).

Additional 17 (17, 39, 153–167) studies were maintained together with those previously included to descriptively explore secondary outcomes of this review.

**Figure 2:**
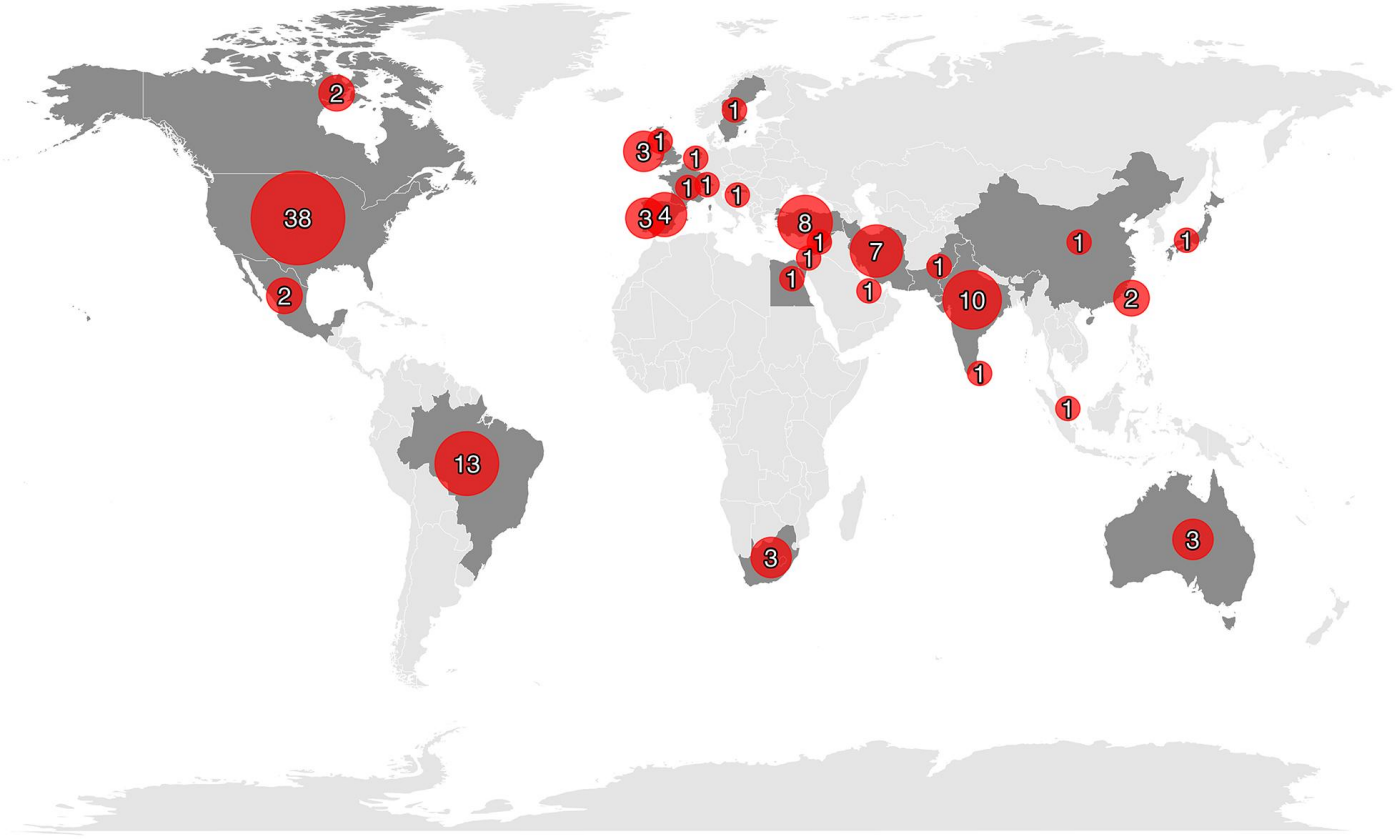
Word map representation of published literature investigating noise levels in the NICU.

According to the primary outcome results, most studies were conducted in the United States (n = 38) and South America (*n* = 13), together accounting for nearly half of the included literature. A detailed geographical distribution is presented in Figure 2.

**Figure 3:**
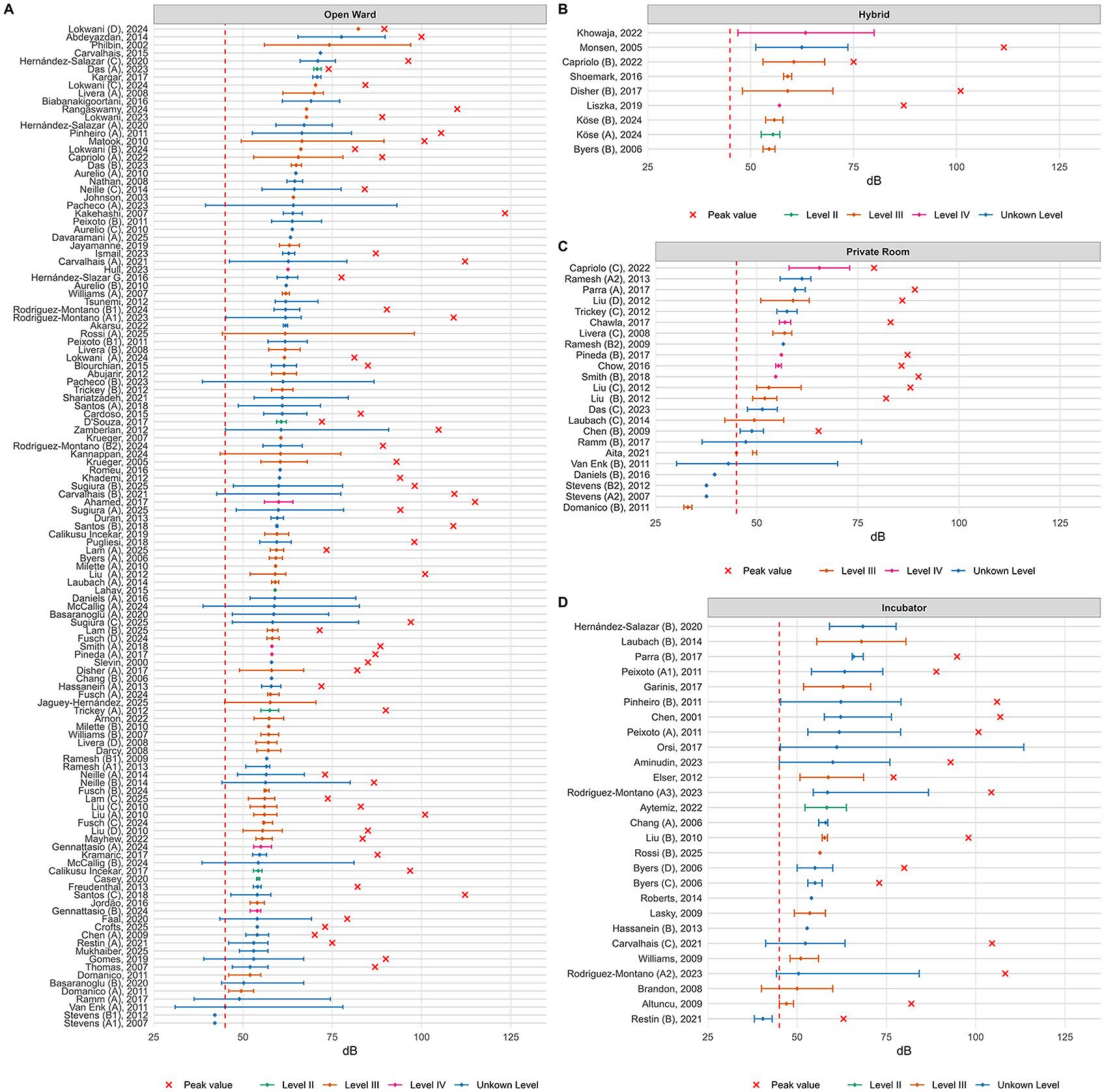
Strip-plot evaluating noise level variation in the NICU, where the red discontinued line indicates reference thresholds defined by the American Academy of Pediatrics (AAP). The figure reports operational ranges and 24 h mean values when available. If 24 h values could not be extracted or were not reported, an operational mean was calculated out of the operational ranges. Noise values in **(A)** Open wards NICUs, **(B)** Hybrid NICUS, **(C)** Private room, and **(D)** Incubator.

When looking at operational levels (both ranges and means) values almost always exceeded the 45 dB threshold recommended by the AAP (Figure 3; Supplementary Table S2, with some even reporting peak noise values above 100 dB.

**Figure 4:**
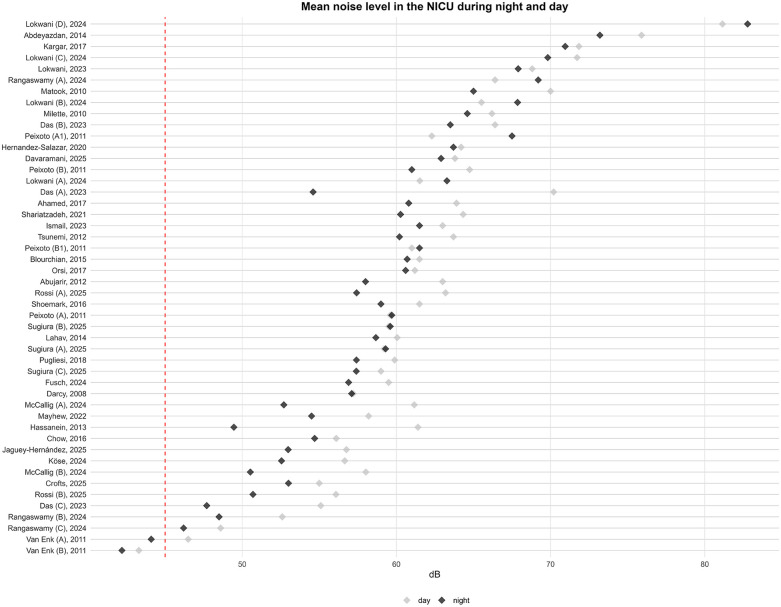
Dot-plot illustrating day vs. night differences in the measured NICU noise environment. The red discontinued line indicates reference thresholds defined by the American Academy of Pediatrics (AAP). Several authors are reported more than once, as their work addresses different: contexts, settings, NICU levels, Hospital, and/or intervention.

Daytime values were tendentially higher than nighttime levels, although both often exceeded recommended thresholds (Figure 4).

**Figure 5:**
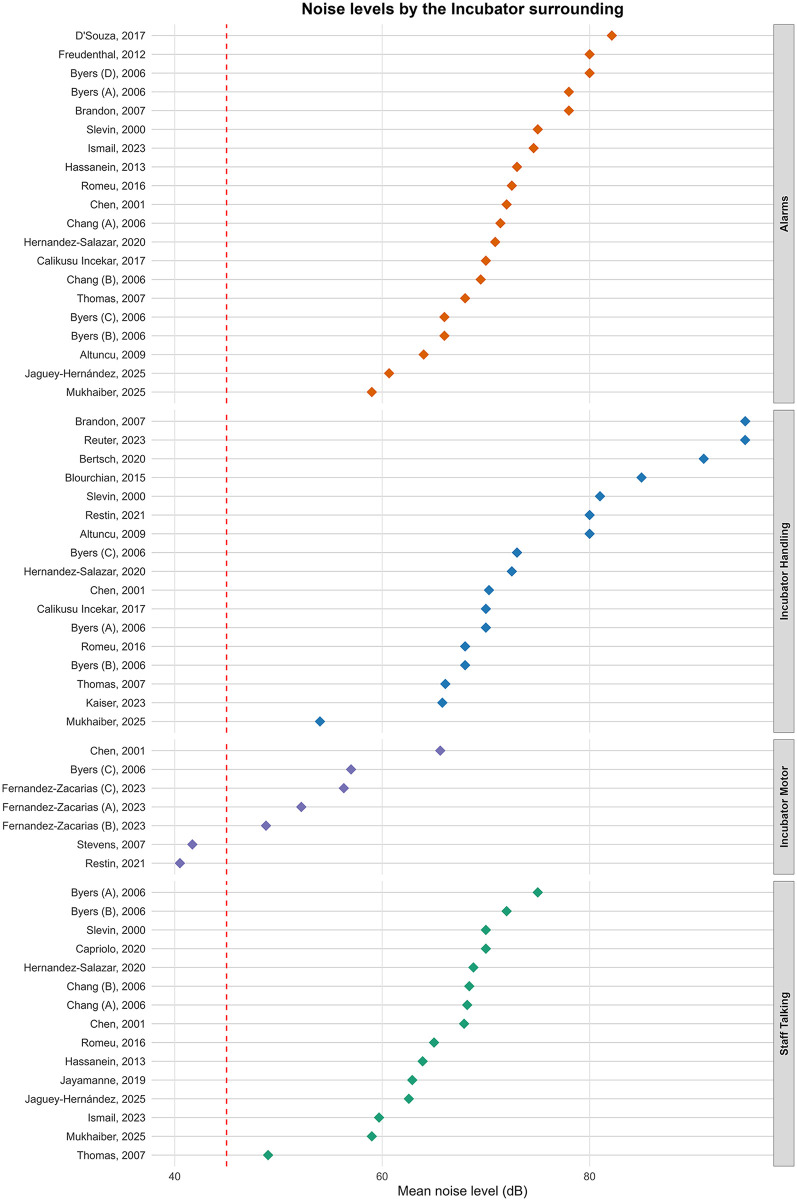
Noise sources organized into four categories: staff talking, alarms, incubator motor, and incubator handling. The red discontinued line indicates reference thresholds defined by the American Academy of Pediatrics (AAP).

Regarding specific noise sources, elevated values were observed across all predefined categories: staff talking, alarms, incubator motor noise, and action on incubator (Figure 5). These findings confirm that NICU soundscapes are shaped by a combination of human activity and mechanical equipment.

**Figure 6:**
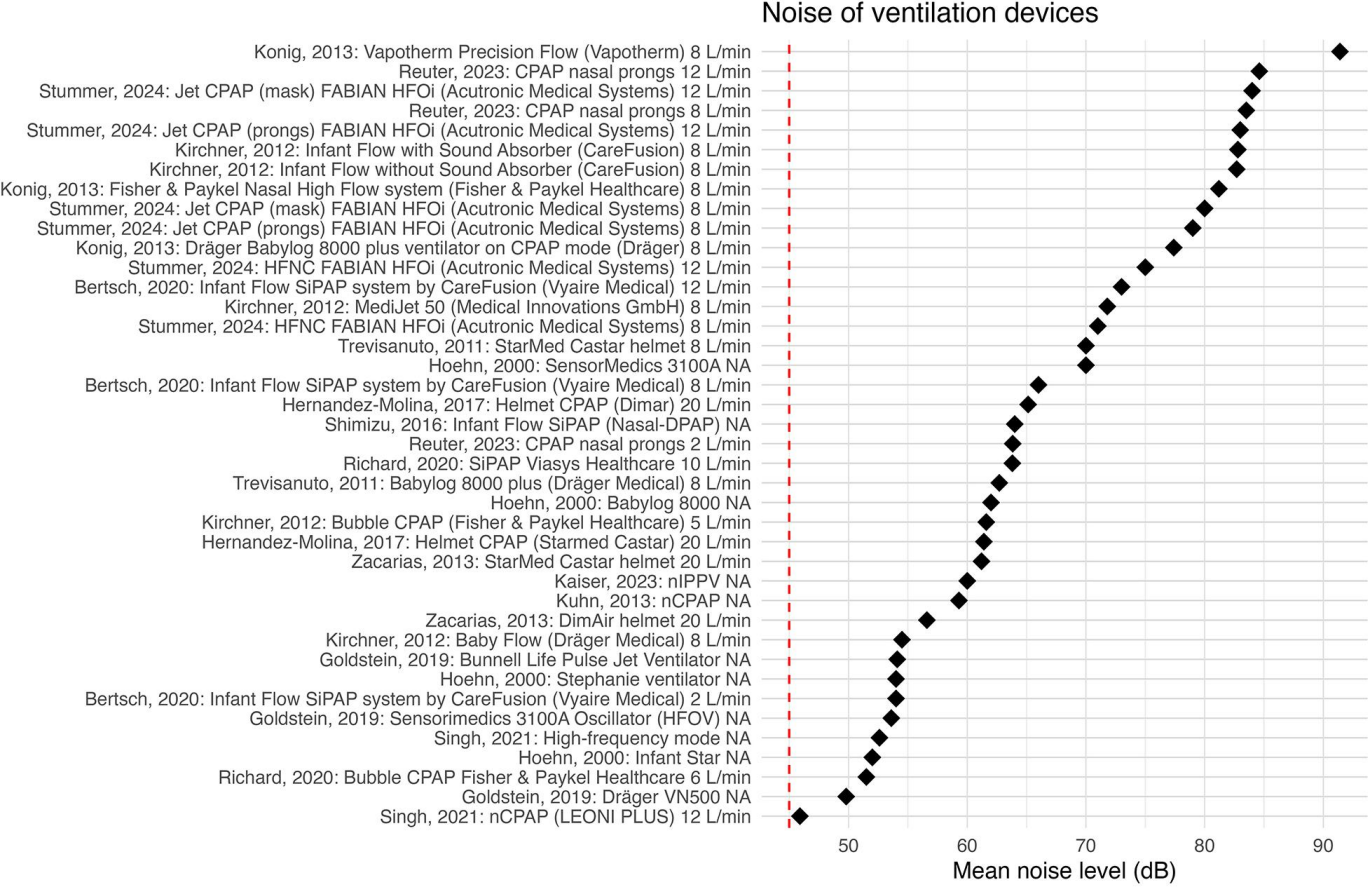
Dot-plot representing noise sources from different ventilation devices. The red discontinued line indicates reference thresholds defined by the American Academy of Pediatrics (AAP).

Among all internal incubator noise sources, respiratory support devices produced the highest values. In the noise spectrum of some continuous positive airway pressure (CPAP) respiratory supports, the strongest amplitudes are just in that frequency area where preterm babies hear most sensitively. In particular, the Vapotherm system was identified as the loudest device, while the LEONI PLUS ventilator exhibited the lowest noise emissions (Figure 6).

**Figure 7:**
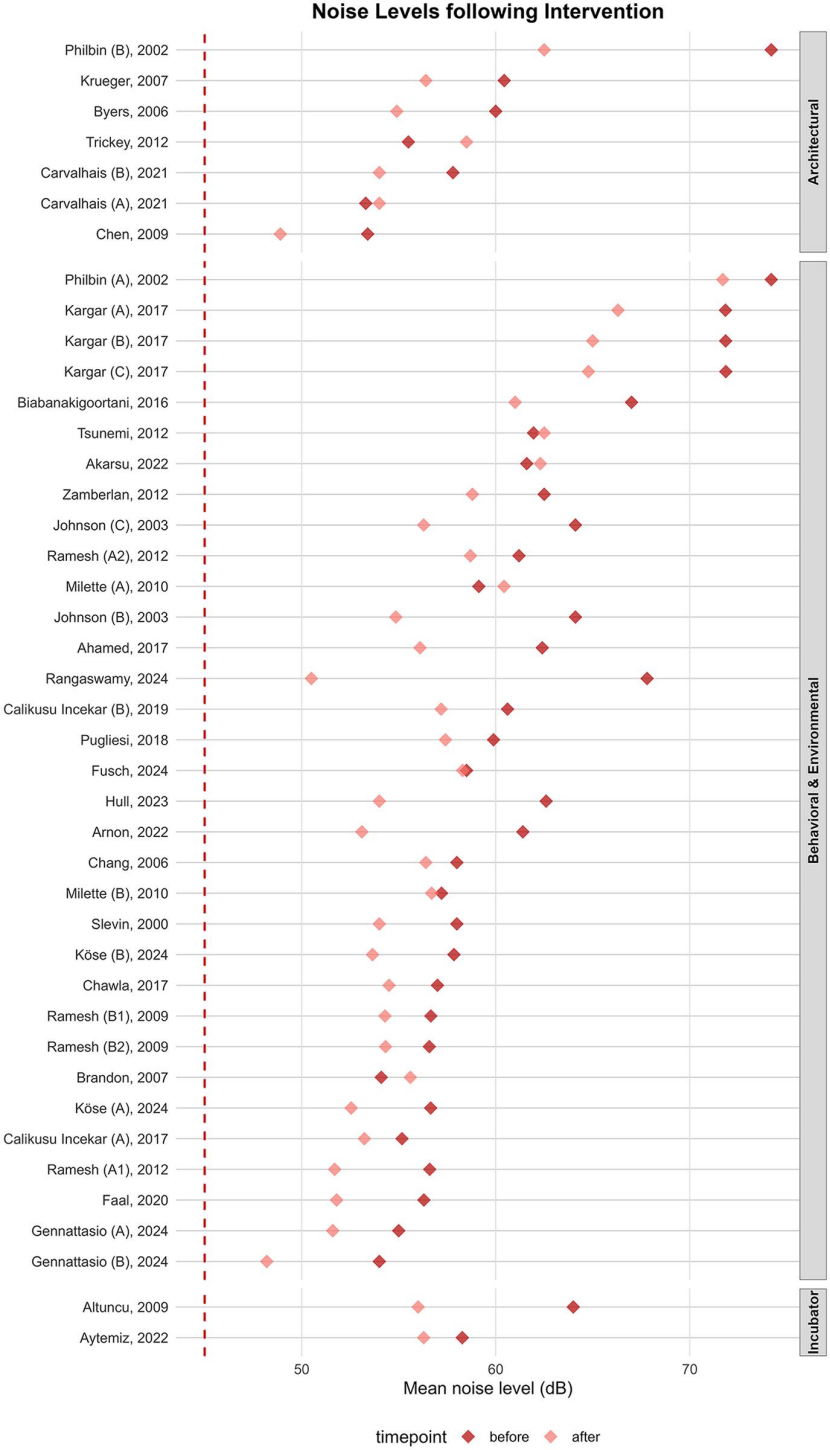
Dot-plot representing NICU noise levels under different interventional and environmental conditions. Some author report different time point as well as different setting, therefore more context information is provided in Supplementary Table S3.

**Figure 8:**
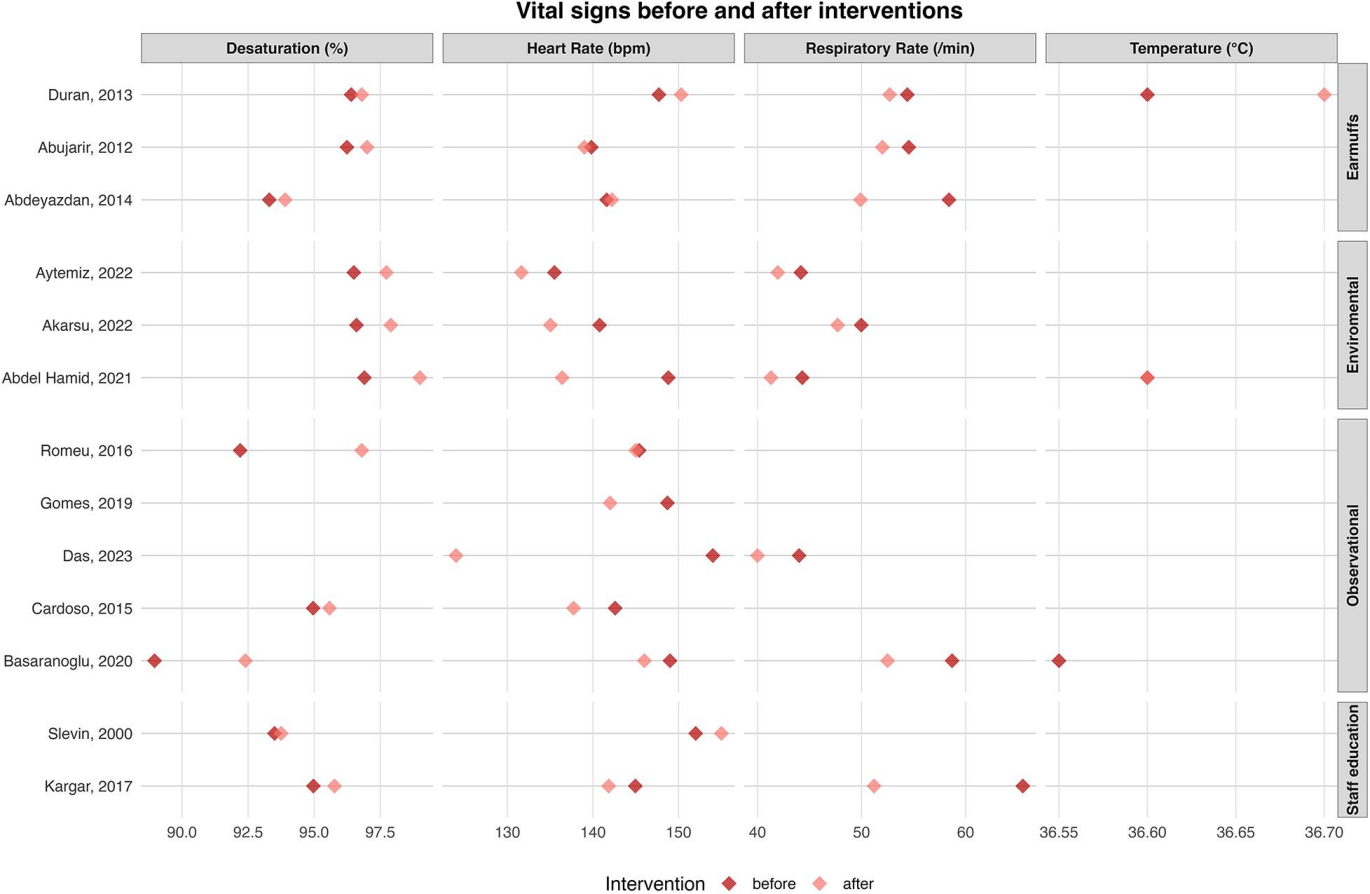
Dot-plot regarding the impact of noise on physiological response by the infant.

Finally, some studies focused on changes in noise level following intervention or comparing settings. More specifically, these studies examined the impact of educational or structural intervention on noise level (pre/post intervention; Figure 7, Supplementary Table S3) or on the physiological response of the infant (Figure 8).

Risk of bias are presented in Supplementary Table S4 and Figure S1. Forty-seven studies had low risk of bias, six had low to moderate risk of bias, 33 had moderate risk of bias, 23 had moderate to high risk of bias, and four had high risk of bias. Moreover, according to the Web of Science journal citation report, 17 studies were considered Q1, 33 were considered Q2, 29 were considered Q3, 23 were considered Q4, while 11 had no quartile expressed yet.

## Discussion

Our study provides a comprehensive summary of the current literature on noise levels in NICUs, highlighting that reported sound levels consistently exceed the limits set by the AAP. This finding warrants critical attention from healthcare professionals and policymakers.

Noise pollution is a pervasive public health issue affecting individuals across the lifespan (14, 34, 55). In hospital settings, excessive noise delays healing, disrupts sleep, elevates stress levels, and increases the risk of medical errors (33).

### Noise in the NICU environment

Understanding and addressing the sources of NICU noise is essential for improving care in this vulnerable population. Our findings confirm, in line with Andy et al. (30), that both external (NICU environment) and internal (incubator) noise sources, frequently exceed WHO limits of 35–40 dB for hospitals (168, 169). The AAP further recommends a maximum of 45 dB during daytime and 35 dB at night (28). Consistent with Busch-Vishniac et al. (170) and de Lima Andrade et al. (13), our review demonstrates that excessive noise levels persist despite architectural and technical improvements (171, 172).

We also found that respiratory support devices contribute to the acoustic environment within the incubator. Although incubators are often considered protective barriers against ambient NICU noise, several studies indicate that internal device-generated sounds may represent a significant source of acoustic exposure for preterm infants. In particular, respiratory support systems such as CPAP and high-flow devices can generate sound levels that exceed recommended NICU noise thresholds and may dominate the acoustic environment inside the incubator. Recent experimental work show that some respiratory modalities, including jet CPAP systems, can generate substantially higher noise levels than other ventilatory strategies, suggesting that the type of respiratory support can directly influence the soundscape experienced by the infant (17, 39, 40).

From a clinical perspective, these findings suggest that respiratory support technologies, while essential for neonatal care, may represent an underrecognized contributor to the overall acoustic burden within incubators. This underscores the importance of considering the acoustic characteristics of respiratory equipment, incubator design, and device placement when developing strategies aimed at improving the sensory environment of preterm infants in NICU settings.

### Physiological and developmental consequences

Although the primary focus of this review was the characterization of the NICU acoustic environment, existing evidence suggests that excessive noise exposure may have relevant implications for preterm infants.

Preterm infants are uniquely sensitive to environmental stressors due to immature regulatory systems. High noise levels disrupt sleep–wake cycles, which are vital for synaptogenesis, neural plasticity, and memory formation, which are core markers of early brain development (35, 173–177). Moreover, fluctuating and excessive sound triggers physiological stress responses including tachycardia, tachypnea, elevated blood pressure, reduced oxygen saturation, hypoxic episodes, apnea, bradycardia, greater oxygen/caloric demands, and altered neuroendocrine and immune function (133, 178–181). Given the vulnerability of the developing auditory and neurological systems in preterm infants, prolonged exposure to high noise levels may also contribute to adverse neurodevelopmental effects. However, current evidence remains heterogeneous, and further longitudinal studies are needed to better clarify the long-term developmental implications of NICU noise exposure. At the same time, strategies aimed at reducing environmental noise should consider the potential trade-off between harmful sound exposure and excessive acoustic deprivation, as the developing auditory system also requires appropriate sensory stimulation for normal maturation.

### Noise reduction strategies in the NICU

Unit-level interventions have shown short-term success in reducing ambient noise. Staff- and parent-centered education programs, often structured within Plan–Do–Study–Act (PDSA) cycles, can reduce average sound levels substantially, as illustrated by a study achieving a drop from 68 dB to 50 dB within one year (179).

Real-time feedback systems such as traffic-light sound meters offer visual cues but tend to lose effectiveness without consistent reinforcement (182). Scheduled “quiet time” blocks and behavioral nudges (e.g., posters, noise champions) are helpful, especially when bundled with orientation checklists and policy tools (49, 112, 183). However, strategies targeting the infant–incubator interface remain under explored (183). Stummer et al. (17) showed that respiratory support devices often generate internal sound levels exceeding both NICU ambient values and AAP thresholds, rendering incubators less protective and occasionally amplifying internal sound.

Passive protective measures such as earmuffs or earplugs have yielded inconsistent results in small-scale trials and may not offer physiological benefit (183). In contrast, simulated active noise control (ANC) systems have demonstrated reductions of up to 14 dB for tonal alarms inside incubators without requiring direct physical contact with the infant (184).

### Balancing noise mitigation and sensory stimulation

Auditory deprivation is an emerging concern. In this context, meaningful auditory input refers to structured, biologically relevant sounds, such as parental voice, gentle speech, or controlled music exposure. Such input may support sensory and neurological development, unlike the unpredictable mechanical noise generated by medical equipment or alarms.

Notably, Pineda et al. (123, 185, 186) found that infants in private NICU rooms had less exposure to language and showed poorer long-term language outcomes, suggesting that over-shielding may also impair neurodevelopment. This underscores the need to balance noise reduction with exposure to developmentally meaningful auditory input, such as maternal voice or music (187).

Therefore, the goal of NICU sound management should not be the elimination of all auditory stimuli, but rather the reduction of harmful environmental noise while preserving developmentally appropriate sensory input.

NICU layout and cohorting strategies can modulate acoustic conditions. A redesign into smaller clinical microsystems at McMaster Children’s Hospital in Canada led to a noise reduction of ∼3–4 dBA in low-acuity areas, though overall levels still exceeded recommended thresholds (77). Reverberation time is another factor: private rooms offer shorter reverberation times than open bays, improving acoustic comfort, but potentially reducing interactive sound exposure (188).

### Evidence gaps and future directions

There is a lack of well-designed clinical trials evaluating noise reduction at the incubator level. Long-term outcomes such as neurodevelopment, growth, hearing, or hospital length of stay in relation to sound exposure remain insufficiently studied. Environmental design studies are rare and often limited to descriptive data. Despite strong evidence for harmful effects of noise, auditory support strategies (e.g., maternal voice playback, music therapy) are rarely integrated into routine NICU protocols, even though preliminary data suggest benefits for autonomic and neurological function (187). Addressing NICU noise will likely require multidisciplinary approaches combining clinical practice changes, architectural design solutions, and innovations in biomedical device engineering.

### Limitation

This review has several limitations that should be considered when interpreting the findings. First, the search strategy was limited to studies published in English, which may have resulted in the exclusion of relevant studies published in other languages. Although multiple major databases were systematically searched, it is possible that some relevant studies indexed in other databases were not captured. In addition, the search strategy was fine-tuned only for the primary outcome of this review; therefore, although information on secondary outcomes was extracted when available, the search may not have comprehensively captured all studies.

Another limitation relates to the heterogeneity of the included studies. The reviewed literature differed considerably in terms of study design, noise measurement methodologies, recording duration, NICU settings, and types of equipment assessed. This variability limited the possibility of performing a quantitative synthesis and required a primarily descriptive approach.

Furthermore, the time frame of the search strategy may affect the interpretation of the findings, as technological advancements in NICU architecture, monitoring systems, and medical equipment have evolved over time and may have influenced acoustic conditions within neonatal intensive care environments.

An additional limitation of this review relates to the descriptive nature of the available literature on NICU noise levels. Because most studies focus on environmental sound measurements and consistently report similar findings, the synthesis of results inevitably reflects a largely convergent body of evidence, which may partially limit the separation between descriptive reporting and interpretative discussion.

Finally, the review protocol was not prospectively registered in PROSPERO. Although the study was conducted following PRISMA reporting guidelines, and performed by trained personal of the MUW (BW).

Despite these limitations, the review provides a comprehensive overview of the current evidence regarding acoustic conditions in NICU environments and highlights important areas for future research and potential strategies to improve the auditory environment for preterm infants.

## Conclusion

Excessive noise in the NICU should be recognized as an important and modifiable environmental factor that may affect the physiological stability and sensory experience of preterm infants. Although the available evidence is largely observational, the consistency of findings across studies indicates that sound levels in NICU environments frequently exceed recommended thresholds.

Based on the current evidence, several practical strategies may help reduce harmful acoustic exposure. These include improving the acoustic performance of incubators, optimizing the design and management of alarm systems, and implementing standardized acoustic monitoring within NICU environments. In addition, targeted engineering solutions aimed at reducing noise generated by respiratory support devices and other medical equipment may represent an important area for technological innovation.

At the same time, efforts to reduce environmental noise should avoid excessive sound attenuation that could limit developmentally meaningful auditory stimulation. Approaches that combine noise reduction with structured sensory input, such as exposure to parental voice or controlled auditory stimulation, may offer a more balanced strategy for supporting early sensory development.

Future research should prioritize well-designed clinical and engineering studies, particularly those evaluating incubator-level interventions, alarm redesign, and continuous acoustic monitoring systems. Such studies will be essential to better understand how modifications of the NICU sound environment may contribute to improving the care and developmental support of preterm infants.

## Data Availability

The original contributions presented in the study are included in the article/Supplementary Material, further inquiries can be directed to the corresponding author.
