# Supplementary material for: What’s that noise—tackling sound pollution in the NICU: a systematic review
Source: Front Pediatr. 2026 Jun 12;14:1798036. doi: 10.3389/fped.2026.1798036 (PMC13303460; doi:10.3389/fped.2026.1798036)
Supplement: Supplementary file 1 [file Table1.docx]

Supplemrntary Material

**ST1 - Search strategy description**

The main topic "*nicu*" representing the population was searched using controlled vocabulary (MeSH, Emtree) and as free text terms in the title, abstract and author keyword fields. Synonyms were collected through reading and prepared for research by using appropriate truncation and a precisely set proximity to broaden the search to ensure the identification of all relevant papers.

Search strategy for the topic "*nicu*" in Embase:

| #1 OR #2 OR #3 OR #4 OR #5 OR #6 OR #7 |
| --- |
| nicu:ti,ab,kw OR nicus:ti,ab,kw |
| ((icu OR icus) NEAR/2 (neonatal* OR newborn* OR baby*)):ti,ab,kw |
| (incubator* NEAR/5 (neonatal* OR newborn* OR infant* OR baby*)):ti,ab,kw |
| ('intensive care medicine*' NEAR/2 (neonatal* OR newborn* OR baby*)):ti,ab,kw |
| ('intensive care department*' NEAR/2 (neonatal* OR newborn* OR baby*)):ti,ab,kw |
| ('intensive care unit*' NEAR/2 (neonatal* OR newborn* OR baby*)):ti,ab,kw |
| 'neonatal intensive care unit'/exp |

The same approach was applied to the second main topic "*noise/sound pollution/level*" which represents exposure. All hierarchically relevant terms and phrases within the controlled vocabulary were carefully reviewed. Appropriate index terms were selected and more than adequately replaced by a free text search in the title/abstract/author keyword/index term field, and further enhanced with synonyms, truncation, and proximity operators.

Search strategy for the topic *"noise/sound pollution/level"* in Embase:

| #9 OR #10 OR #11 OR #12 |
| --- |
| decibel*:ti,ab,kw,de |
| (sound* NEAR/5 (level* OR measur* OR detect* OR intensit* OR identificat* OR environment* OR exposur* OR exposed* OR electric* OR reduc* OR assess* OR manage* OR evaluat* OR source* OR pollution*)):ti,ab,kw,de |
| (acoustic* NEAR/5 (level* OR measur* OR detect* OR intensit* OR identificat* OR environment* OR exposur* OR exposed* OR electric* OR reduc* OR assess* OR manage* OR evaluat* OR source* OR pollution*)):ti,ab,kw,de |
| (noise* NEAR/5 (level* OR measur* OR detect* OR intensit* OR identificat* OR environment* OR exposur* OR exposed* OR electric* OR reduc* OR assess* OR manage* OR evaluat* OR source* OR pollution*)):ti,ab,kw,de |

To ensure a comprehensive and appropriate search strategy, no additional restrictions were applied after combining the two main topics, apart from limiting the results to publications from the year 2000 onward.

The impact on result quality was continuously evaluated at every stage of the search strategy development. In addition, ongoing text analysis was conducted through careful reading and a corresponding critical reflection on the search strategy.

The search strategy for Embase was then transferred to all other database.

**Table S1 - JBI Critical Appraisal Checklist for analytical cross sectional studies**

Reviewer ______________________________________ Date_______________________________

Author_______________________________________ Year_________ Record Number_________

|  | Yes | No | Unclear | Not applicable |
| --- | --- | --- | --- | --- |
| 1. Were the acoustic measurement criteria clearly defined (e.g., devices, units)? | □ | □ | □ | □ |
| 1. Was the NICU setting described in detail? | □ | □ | □ | □ |
| 1. Was the exposure (i.e., noise) measured in a valid and reliable way (e.g., how many timepoints did they measure)? | □ | □ | □ | □ |
| 1. Were objective, standard criteria used for measurement of noise (i.e., quality of measurement device)? | □ | □ | □ | □ |
| 1. Were confounding factors identified (e.g., staff talking during measurement)? | □ | □ | □ | □ |
| 1. Were strategies to deal with confounding factors stated (e.g., informed staff)? | □ | □ | □ | □ |
| 1. Were the outcomes measured in a valid and reliable way (summary of Q1, 3, and 4)? | □ | □ | □ | □ |
| 1. Was appropriate statistical analysis used (i.e., minimum requirement of noise measurement = mean and SD and/or their change in different situations, such as time of day)? | □ | □ | □ | □ |

Comments (Including reason for exclusion)

**Table S2: Extra information on measurement condition for operational level related to Figure 3**

| Byers (A) | 2006 | Orlando | USA | Level III | Open ward | Bedside |
| --- | --- | --- | --- | --- | --- | --- |
| Byers (B) | 2006 | Orlando | USA | Level III | Hybrid | Bedside |
| Byers (C) | 2006 | Orlando | USA |  | Incubator | Incubator |
| Byers (D) | 2006 | Orlando | USA |  | Incubator | Open warmer |
| Capriolo (A) | 2022 | Maryland | USA | Level III | Open ward | Bedside |
| Capriolo (B) | 2022 | Maryland | USA | Level III | Hybrid | Bedside |
| Capriolo (C) | 2022 | Maryland | USA | Level IV | Private Room | Bedside (SFR) |
| Domanico (A) | 2011 | Huntington | USA | Level III | Open ward | Near incubator |
| Domanico (B) | 2011 | Huntington | USA | Level III | Private Room | Near incubator (SFR) |
| Trickey (A) | 2012 | Texas | USA | Level II | Open ward | Patient room |
| Trickey (B) | 2012 | Texas | USA | Level III | Open ward | Patient room |
| Trickey (C) | 2012 | Texas | USA |  | Private Room | Isolation room |
| Abdeyazdan | 2014 | Isfahan | Iran |  | Open ward | Near incubator |
| Abujarir | 2012 | Doha | Qatar | Level III | Open ward | Incubator |
| Ahamed | 2017 | Bronx (NY) | USA | Level IV | Open ward | Near incubator |
| Aita | 2021 | Montreal | Canada | Level III | Private Room | Incubator |
| Akarsu | 2022 | Istanbul | Turkey |  | Open ward | Outside incubator |
| Altuncu | 2009 | Istanbul | Turkey | Level III | Incubator | Inside incubator |
| Aminudin | 2023 | Dublin | Ireland |  | Incubator | Inside incubator |
| Arnon | 2022 | Kfar Saba | Israel | Level III | Open ward | Room environment |
| Aurelio (A) | 2010 | Santa Maria | Brazil |  | Open ward | Intensive care room |
| Aurelio (B) | 2010 | Santa Maria | Brazil |  | Open ward | Intermediate care room |
| Aurelio (C) | 2010 | Santa Maria | Brazil |  | Open ward | Isolation room |
| Aytemiz | 2022 | Istanbul | Turkey | Level II | Incubator | Inside incubator |
| Basaranoglu (A) | 2020 | Van | Turkey |  | Open ward | Section 1 (high activity area) |
| Basaranoglu (B) | 2020 | Van | Turkey |  | Open ward | Section 2 (low activity area) |
| Biabanakigoortani | 2016 | Tehran | Iran |  | Open ward | NICU environment |
| Blourchian | 2015 | Rasht | Iran |  | Open ward | NICU environment |
| Brandon | 2008 | Durham (NC) | USA | Level III | Incubator | Inside incubator |
| Calikusu Incekar (A) | 2017 | Istanbul | Turkey | Level II–III | Open ward | NICU environment |
| Calikusu Incekar (B) | 2019 | Istanbul | Turkey | Level III | Open ward | NICU environment |
| Cardoso | 2015 | Curitiba | Brazil |  | Open ward | Inside and outside incubator |
| Carvalhais (A) | 2021 | Porto | Portugal |  | Open ward | Workstation (room A1.1) |
| Carvalhais (B) | 2021 | Porto | Portugal |  | Open ward | Workstation (room A1.2) |
| Carvalhais (C) | 2021 | Porto | Portugal |  | Incubator | Inside incubator |
| Casey | 2020 | Kingston | Canada | Level II–III | Open ward | Bays (A–C) |
| Chang (A) | 2006 | Tainan | Taiwan |  | Incubator | Inside incubator |
| Chang (B) | 2006 | Tainan | Taiwan |  | Open ward | Radiant heated bed |
| Chawla | 2017 | Detroit (MI) | USA | Level III–IV | Private Room | Patient room |
| Chen | 2001 | Tainan | Taiwan |  | Incubator | Inside incubator |
| Chen (A) | 2009 | Taichung | Taiwan |  | Open ward | Open space |
| Chen (B) | 2009 | Taichung | Taiwan |  | Private Room | Bedside |
| Chow | 2016 | Ann Arbor (MI) | USA | Level IV | Private Room | Bedside recording |
| Crofts | 2025 |  | England |  | Open ward |  |
| Daniels (A) | 2016 | Columbus (OH) | USA |  | Open ward | Bedside (radiant warmer) |
| Daniels (B) | 2016 | Columbus (OH) | USA |  | Private Room | Bedside (crib) |
| Darcy | 2008 | Mid-Atlantic (urban hospitals) | USA | Level III | Open ward | Central NICU area (ambient) |
| Das (A) | 2023 | Northeast India | India | Level II | Open ward | NICU environment |
| Das (B) |  |  |  | Level lll | Open ward | NICU |
| Das (C) | 2023 | Northeast India | India |  | Private Room | KMC area |
| Davaramani (A) | 2025 |  | India |  | Open ward |  |
| Disher (A) | 2017 | Halifax | Canada | Level III | Open ward | Care areas (NICU 1,2,3) |
| Disher (B) | 2017 | Halifax | Canada | Level lll | Hybrid | Bedside, nursing station, private |
| Domanico | 2011 | Huntington (WV) | USA | Level III | Open ward | Near nursing station / entry / remote sites) |
| D'Souza | 2017 | Udupi (Karnataka) | India | Level II-III | Open ward | Acute NICU (central area, blocks) |
| Duran | 2012 | Edirne | Turkey |  | Open ward | Inside and outside incubator |
| Elser | 2012 | Southeast USA | USA | Level III | Incubator | Inside incubator (near head) |
| Faal | 2020 | Birjand | Iran |  | Open ward | NICU environment |
| Freudenthal | 2012 | Groningen | Netherlands |  | Open ward | Ward center |
| Fusch (A) | 2024 | Hamilton (ON) | Canada | Level III | Open ward | Pod A (high acuity) |
| Fusch (B) | 2024 | Hamilton (ON) | Canada | Level III | Open ward | Pod B (high acuity) |
| Fusch (C) | 2024 | Hamilton (ON) | Canada | Level III | Open ward | Pod D (low acuity) |
| Fusch (D) | 2024 | Hamilton (ON) | Canada | Level III | Open ward | Pod E (low acuity) |
| Hernández-Slazar G | 2016 | Santa Marta | Colombia |  | Open ward | NICU environment |
| Garinis | 2017 | Portland (OR) | USA | Level III | Incubator | Inside incubator (dosimeter near head) |
| Gennattasio (A) | 2024 | New York (NY) | USA | Level IV | Open ward | NICU acute |
| Gennattasio (B) | 2024 | New York (NY) | USA | Level IV | Open ward | NICU Stepdown |
| Gomes | 2019 | São Paulo | Brazil |  | Open ward | Near incubator/crib (per infant) |
| Hassanein (A) | 2013 | Cairo | Egypt |  | Open ward | NICU general |
| Hassanein (B) | 2013 | Cairo | Egypt |  | Incubator | Inside incubator |
| Hernández-Salazar (A) | 2020 | San Luis Potosí | Mexico |  | Open ward | Near incubator |
| Hernández-Salazar (B) | 2020 | San Luis Potosí | Mexico |  | Incubator | Periauricular (20 cm) |
| Hernández-Salazar (C) | 2020 | San Luis Potosí | Mexico |  | Open ward | Near incubator |
| Hull | 2023 | Texas | USA | Level IV | Open ward | Near infant ear |
| Ismail | 2023 | eThekwini District | South Africa |  | Open ward | Central location |
| Jaguey-Hernández | 2025 | Hidalgo | Mexico | Level III | Open ward | Near incubator (30 cm) |
| Jayamanne | 2019 | Colombo | Sri Lanka | Level III | Open ward | Central area |
| Johnson | 2003 | Delaware | USA | Level III | Open ward | Multiple zones |
| Jordão | 2016 | Ribeirão Preto | Brazil | Level III | Open ward | Near incubator |
| Kakehashi | 2007 | São Paulo | Brazil |  | Open ward | Central NICU |
| Kannappan | 2024 | Mangalore | India | Level III | Open ward | NICU |
| Kargar | 2017 | Shiraz | Iran |  | Open ward | NICU |
| Khademi | 2011 | Mashhad | Iran |  | Open ward | ICU |
| Khowaja | 2022 | Karachi | Pakistan | Level IV | Hybrid | Open bay + Isolation Room |
| Köse (A) | 2024 | Konya | Turkey | Level ll | Hybrid | Second level NICU |
| Köse (B) | 2024 | Konya | Turkey | Level lll | Hybrid | Third level NICU |
| Kramarić | 2017 | Osijek | Croatia |  | Open ward | Near incubator |
| Krueger | 2005 | Florida | USA | Level lll | Open ward | Multiple locations |
| Lahav | 2015 | Boston | USA | Level II | Open ward | Central NICU |
| Lam (A) | 2025 | Singapore | Singapore | Level III | Open ward | NICU-A |
| Lam (B) | 2025 | Singapore | Singapore | Level III | Open ward | NICU-B |
| Lam (C) | 2025 | Singapore | Singapore | Level III | Open ward | Binaural, HD-A |
| Lasky | 2009 | Houston | USA | Level III | Incubator | Inside bed (≤30 cm from ear) |
| Laubach (A) | 2014 | Honolulu | USA | Level III | Open ward | Open-bay pods |
| Laubach (B) | 2014 | Honolulu | USA | Level III | Incubator | Inside isolette |
| Laubach (C) | 2014 | Honolulu | USA | Level III | Private Room | Patient room (occupied) |
| Liszka | 2019 | St. Louis | USA | Level IV | Hybrid | NICU |
| Liu (A) | 2010 | Fort Myers | USA | Level III | Open ward | Bedside |
| Liu (B) | 2010 | Fort Myers | USA | Level III | Incubator | Incubator |
| Liu (C) | 2010 | Fort Myers | USA | Level III | Open ward | Staff work area |
| Liu (D) | 2010 | Fort Myers | USA | Level III | Open ward | Central entry area |
| Liu (A) | 2012 | Fort Myers | USA | Level III | Open ward | bedside |
| Liu (B) | 2012 | Fort Myers | USA | Level III | Private Room | Unoccupied room |
| Liu (C) | 2012 | Fort Myers | USA | Level III | Private Room | SFR room air |
| Liu (D) | 2012 | Fort Myers | USA | Level III | Private Room | SFR with PPS (HFNC/BCPAP/VENT) |
| Livera (A) | 2008 | Bangalore | India | Level III | Open ward | Ventilator room |
| Livera (B) | 2008 | Bangalore | India | Level III | Open ward | Stable room |
| Livera (C) | 2008 | Bangalore | India | Level III | Private Room | Isolation room |
| Livera (D) | 2008 | Bangalore | India | Level III | Open ward | Pre-term room |
| Lokwani | 2023 | Jodhpur | India | Level III | Open ward | NICU block |
| Lokwani (A) | 2024 | Jodhpur | India | Level III | Open ward | NICU PH1 |
| Lokwani (B) | 2024 | Jodhpur | India | Level III | Open ward | NICU PH2 |
| Lokwani (C) | 2024 | Jodhpur | India | Level III | Open ward | NICU GH1 |
| Lokwani (D) | 2024 | Jodhpur | India | Level III | Open ward | NICU GH2 |
| Matook | 2010 | Providence | USA | Level III | Open ward | NICU bays |
| Mayhew | 2022 | Ottawa | Canada | Level III | Open ward | Room A |
| McCallig (A) | 2024 | Sligo | Ireland |  | Open ward | Area 1 (nurses station) |
| McCallig (B) | 2024 | Sligo | Ireland |  | Open ward | Area 2 (near cots) |
| Milette (A) | 2010 | Montreal | Canada | Level III | Open ward | Intensive care room (ICC) |
| Milette (B) | 2010 | Montreal | Canada | Level III | Open ward | Intermediate care room (IMC) |
| Monsen | 2005 | Linkoping | Sweden |  | Hybrid | Central room (ceiling-mounted meter) |
| Mukhaiber | 2025 | Damascus | Syria |  | Open ward | Critical care room (incubator room) |
| Nathan | 2008 | Cape Metropole | South Africa |  | Open ward | NICU rooms (central ceiling measurement) |
| Neille (A) | 2014 | Johannesburg | South Africa |  | Open ward | Hospital A |
| Neille (B) | 2014 | Johannesburg | South Africa |  | Open ward | Hospital B |
| Neille (C) | 2014 | Johannesburg | South Africa |  | Open ward | Hospital C |
| Orsi | 2017 | Sao Paulo | Brazil |  | Incubator | Inside incubator (20 cm from ear) |
| Pacheco (A) | 2023 | Niteroi | Brazil |  | Open ward | ICU (central measurement) |
| Pacheco (B) | 2023 | Niteroi | Brazil |  | Open ward | Intermediate unit (IU) |
| Parra (A) | 2017 | Grenoble | France |  | Private Room | Outside incubator (room center) |
| Parra (B) | 2017 | Grenoble | France |  | Incubator | Inside incubator (near infant ear) |
| Peixoto (A) | 2011 | Sao Paulo | Brazil |  | Incubator | Inside incubator (Room A) |
| Peixoto (A1) | 2011 | Sao Paulo | Brazil |  | Incubator | Inside incubator (Room B) |
| Peixoto (B) | 2011 | Sao Paulo | Brazil |  | Open ward | NICU room A (center of room) |
| Peixoto (B1) | 2011 | Sao Paulo | Brazil |  | Open ward | NICU room B (center of room) |
| Philbin | 2002 | Camden, NJ | USA | Level III | Open ward | Single bed space (naïve nursery) |
| Pineda (A) | 2017 | St. Louis, MO | USA | Level IV | Open ward | NICU open ward (near infant, LENA device) |
| Pineda (B) | 2017 | St. Louis, MO | USA | Level IV | Private room | NICU private room (near infant, LENA device) |
| Pinheiro (A) | 2011 | Sao Paulo | Brazil |  | Open ward | NICU room (central spatial average) |
| Pinheiro (B) | 2011 | Sao Paulo | Brazil |  | Incubator | Inside incubator (20 cm from ear) |
| Pugliesi | 2018 | Sao Paulo | Brazil |  | Open ward |  |
| Ramesh (A1) | 2012 | Bangalore | India |  | Open ward | Preterm room |
| Ramesh (A2) | 2012 | Bangalore | India |  | Private room | Isolation room |
| Ramesh (B1) | 2009 | Bangalore | India |  | Open ward | Preterm room |
| Ramesh (B2) | 2009 | Bangalore | India |  | Private room | Isolation room |
| Ramm (A) | 2017 | Adelaide | Australia |  | Open ward | NICU open plan |
| Ramm (B) | 2017 | Adelaide | Australia |  | Private room | NICU pod |
| Rangaswamy | 2024 | Shimoga | India | Level III | Open ward | NICU central cubicle (Baseline) |
| Restin (A) | 2021 | Zurich | Switzerland |  | Open ward | NICU near incubator |
| Restin (B) | 2021 | Zurich | Switzerland |  | Incubator | Inside incubator (10–15 cm from head) |
| Roberts | 2014 | Melbourne | Australia |  | Incubator | Near infant (external auditory meatus) |
| Rodriguez-Montano (A1) | 2023 | Cadiz | Spain |  | Open ward | NICU central area |
| Rodriguez-Montano (A2) | 2023 | Cadiz | Spain |  | Incubator | Inside incubator (Giraffe Omnibed) |
| Rodriguez-Montano (A3) | 2023 | Cadiz | Spain |  | Incubator | Inside incubator (Ohio Care Plus 3000) |
| Rodriguez-Montano (B1) | 2024 | Cadiz | Spain |  | Open ward | NICU (HUPM large room) |
| Rodriguez-Montano (B2) | 2024 | Huelva | Spain |  | Open ward | NICU (HJRJ smaller room) |
| Romeu | 2016 | Barcelona | Spain |  | Open ward | Incubator + NICU |
| Rossi (A) | 2025 | Genova | Italy | LevelIII | Open ward | NICU bedside |
| Rossi (B) | 2025 | Genova | Italy | Level III | Incubator | Inside incubator |
| Santos (A) | 2018 | Porto | Portugal |  | Open ward | NICU A (mixed areas) |
| Santos (B) | 2018 | Porto | Portugal |  | Open ward | NICU B (mixed areas) |
| Santos (C) | 2018 | Porto | Portugal |  | Open ward | NICU C (mixed areas) |
| Shariatzadeh | 2021 | Ardabil | Iran |  | Open ward | NICU (multiple stations) |
| Shoemark | 2016 | Melbourne | Australia | Level III | Hybrid | NICU bedside (near infant head) |
| Slevin | 2000 | Dublin | Ireland |  | Open ward | NICU bedside (open care tables) |
| Smith (A) | 2018 | St. Louis | USA | Level IV | Open ward | NICU open pod |
| Smith (B) | 2018 | St. Louis | USA | Level IV | Private room | NICU single room |
| Stevens (A1) | 2007 | Sioux Falls | USA |  | Open ward | NICU bedside (traditional unit) |
| Stevens (A2) | 2007 | Sioux Falls | USA |  | Private room | NICU bedside (single-family room) |
| Stevens (B1) | 2012 | Sioux Falls | USA |  | Open ward | NICU bedside |
| Stevens (B2) | 2012 | Sioux Falls | USA |  | Private room | NICU bedside |
| Sugiura (A) | 2025 |  | Japan |  | Open ward | NICU bedside (loud bays) |
| Sugiura (B) | 2025 |  | Japan |  | Open ward | NICU bedside (intermediate bays) |
| Sugiura (C) | 2025 |  | Japan |  | Open ward | NICU bedside (quiet bays) |
| Thomas | 2007 |  | USA |  | Open ward | NICU + incubator (event-based measurements) |
| Tsunemi | 2012 | São Paulo | Brazil |  | Open ward | NICU (3-point measurement, clinical environment) |
| Van Enk (A) | 2011 | Kalamazoo | USA |  | Open ward | NICU bedside (short-term measurements) |
| Van Enk (B) | 2011 | Kalamazoo | USA |  | Private room | NICU bedside (short-term measurements) |
| Williams | 2009 | Houston | USA | Level III | Incubator | NICU incubator (inside, near infant ear) |
| Williams (A) | 2007 | Houston | USA | Level lll | Open ward | NICU A |
| Williams (B) | 2007 | Houston | USA | Level lll | Open ward | NICU B |
| Zamberlan | 2012 | Ribeirão Preto | Brazil |  | Open ward | NICU room (central area, 24h measurement) |

**Table S3: Extra information related to Figure 7**

| Author | Year | NICU Level | Location/Condition/position | Intervention Type | Interventions |
| --- | --- | --- | --- | --- | --- |
| Byers | 2006 | Level III | Bedside | Architectural | Renovation |
| Trickey | 2012 | Level II | Patient room | Architectural | Construction level ll & lll |
| Ahamed | 2017 | Level IV | Near incubator | Behavioral + environmental | Education, alarm reduction, quiet time, noise monitorBehavioral + environmental (NeuroN-QI) |
| Akarsu | 2022 |  | Outside incubator | Enviromental | NICU ambient noise |
| Altuncu | 2009 | Level III | Inside incubator | Incubator | Sound absorbing panel |
| Arnon | 2022 | Level III | Room environment | Behavioural | Music Therapy |
| Aytemiz | 2022 | Level II | Inside incubator | Incubator | Cover reduces internal incubator noise by ~2 dB |
| Biabanakigoortani | 2016 |  | NICU environment | Behavioral | Educational (peer training) |
| Brandon | 2007 | Level III | Inside incubator | Behavioral + environmental | Some interventions increased noise |
| Calikusu Incekar (A) | 2017 | Level II–III | NICU environment | Behavioral | Educational (noise control training) |
| Calikusu Incekar (B) | 2019 | Level III | NICU environment | Behavioral | Educational (noise control training reduced noise over time (6-week follow-up) |
| Carvalhais (A) | 2021 |  | Workstation (room A1.1) | Architectural | Layout change without significant noise reduction |
| Carvalhais (B) | 2021 |  | Workstation (room A1.2) | Architectural | Layout change without significant noise reduction |
| Chang | 2006 |  | Inside incubator | Behavioral + environmental | SPN reduced 630→185 events/day |
| Chawla | 2017 | Level III–IV | Patient room (multipod) | Behavioral + environmental | Multiphase QI (education + quiet time + alarm reduction + visual feedback) |
| Chen | 2009 |  | Open space | Architectural | NICU design (open vs enclosed) Open space louder than enclosed |
| Faal | 2020 |  | NICU environment | Behavioral + environmental | Training + visual signs |
| Fusch | 2024 | Level III | Pod A (high acuity) | Behavioral + environmental | Staff activity, handover, workload-driven noise |
| Gennattasio (A) | 2024 | Level IV | NICU acute | Behavioral + environmental | Education + equipment + HUSH |
| Gennattasio (B) | 2024 | Level IV | NICU Stepdown | Behavioral + environmental | Education + equipment + HUSH |
| Hull | 2023 | Level IV | Near infant ear | Behavioral | Educational |
| Johnson (B) | 2003 | Level III | Multiple zones | Behavioral + environmental | Post 1 week |
| Johnson (C) | 2003 | Level III | Multiple zones | Behavioral + environmental | Post stabilization period |
| Kargar (A) | 2017 |  | NICU | Behavioral + environmental | 1week |
| Kargar (B) | 2017 |  | NICU | Behavioral + environmental | 2week |
| Kargar (C) | 2017 |  | NICU | Behavioral + environmental | 3week |
| Köse (A) | 2024 | Level ll | Second level NICU | Behavioral + environmental | Contextualized intervention program |
| Köse (B) | 2024 | Level lll | Third level NICU | Behavioral + environmental | Contextualized intervention program |
| Krueger | 2007 | Level lll | NICU | Architectural | Structural enviroment |
| Milette (A) | 2010 | Level III | Intensive care room (ICC) | Behavioral | Educational program (NAEP) |
| Milette (B) | 2010 | Level III | Intermediate care room (IMC) | Behavioral | Educational program (NAEP) |
| Philbin (A) | 2002 | Level III | Single bed space (naïve nursery) | Behavioural | Staff behavior (baseline), equipment, facility noise |
| Philbin (B) | 2002 | Level III | Single bed space (after staff change) | Architectural | Facility renovation, staff behavior, equipment, care activity |
| Pugliesi | 2018 |  | Inside incubator | Behavioral + environmental | Quiet time intervention, staff activity, handling reduction, alarms, environmental noise |
| Ramesh (A1) | 2012 |  | Preterm room | Behavioral + environmental | Staff behavior, alarms, equipment, activity |
| Ramesh (A2) | 2012 |  | Isolation room | Behavioral + environmental | Staff behavior, alarms, equipment, activity |
| Ramesh (B1) | 2009 |  | Preterm room | Behavioral + environmental | Staff behavior, alarms, equipment, activity |
| Ramesh (B2) | 2009 |  | Isolation room | Behavioral + environmental | Staff behavior, alarms, equipment, activity |
| Rangaswamy | 2024 | Level III | NICU central cubicle (Baseline) | Behavioural | PDSA cycle 1 |
| Slevin | 2000 |  | NICU bedside (open care tables) | Behavioural | Quiet protocol (reduced light, noise, handling, staff activity) |
| Tsunemi | 2012 |  | NICU (3-point measurement, clinical environment) | Behavioural | Educational program |
| Zamberlan | 2012 |  | NICU room (central area, 24h measurement) | Behavioral + environmental | Staff behavior, alarms, equipment, activity |

**Table S4: Risk of Bias**

| **Study** | **IF Q1-Q4** | **Q1_Inclusion**  **Criteria** | **Q2_Subjects**  **Setting** | **Q3_Exposure**  **Valid** | **Q4_Objective**  **Criteria** | **Q5_Confounders**  **Identified** | **Q6_Confounders**  **Addressed** | **Q7_Outcomes**  **Valid** | **Q8_Stats**  **Appropriate** | **Overall_RoB** |
| --- | --- | --- | --- | --- | --- | --- | --- | --- | --- | --- |
| Abdeyazdan 2014 | 3 | 0 | 0 | 0 | 1 | 0 | 0 | 0 | 0 | High |
| Abujarir 2012 | 3 | 1 | 1 | 0 | 1 | 0 | 0 | 0 | 1 | Moderate |
| Ahamed 2017 | 4 | 1 | 1 | 1 | 1 | 1 | 1 | 1 | 1 | Low |
| Aita 2021 | 1 | 1 | 1 | 1 | 1 | 1 | 1 | 1 | 1 | Low |
| Akarsu 2022 | 4 | 1 | 1 | 1 | U | 1 | 0 | U | 1 | Moderate/High |
| Altuncu 2009 | 3 | 1 | 0 | 0 | 1 | 1 | 1 | U | 0 | Moderate |
| Aminudin 2023 | 2 | 1 | 1 | 1 | 1 | 1 | 1 | 1 | 1 | Low |
| Arnon 2022 | 2 | 1 | 1 | 1 | U | 1 | 1 | 1 | 1 | Low |
| Aurelio 2010 | 2 | 1 | 1 | 1 | 1 | 1 | 1 | 1 | 0 | Low |
| Aytemiz 2022 | 2 | U | 1 | U | 0 | 1 | 1 | U | 1 | Moderate/High |
| Basaranoglu 2020 | 0 | 1 | 0 | 1 | 1 | 1 | 0 | 1 | 1 | Moderate |
| Biabanakigoortani 2016 | 3 | 1 | 0 | 1 | U | 0 | 0 | U | 1 | Moderate |
| Blourchian 2015 | 4 | 0 | 0 | 1 | U | 1 | 0 | U | 1 | Moderate |
| Brandon 2008 | 2 | 1 | 1 | 1 | 1 | 1 | 1 | 1 | 1 | Low |
| Byers 2006 | 4 | 1 | 1 | 1 | 1 | 1 | 1 | 1 | 1 | Low |
| Calikusu Incekar 2017 | 3 | 1 | 1 | 1 | 1 | 1 | 1 | 1 | 1 | Low |
| Calikusu Incekar 2019 | 3 | 1 | 1 | 1 | 1 | 1 | 1 | 1 | 1 | Low |
| Capriolo 2022 | 3 | 1 | 1 | 1 | 0 | 1 | 1 | 0 | 1 | Moderate |
| Cardoso 2015 | 2 | 1 | 0 | 1 | 1 | 0 | 0 | 1 | 1 | Moderate |
| Carvalhais 2015 | 2 | 1 | 1 | 1 | 1 | 0 | 0 | 1 | 1 | Low |
| Carvalhais 2021 | 4 | 1 | 1 | U | 1 | 0 | 0 | U | 1 | Moderate |
| Casey 2020 | 2 | 1 | 1 | 1 | 1 | 1 | 1 | 1 | 1 | Low |
| Chang 2006 | 4 | 1 | 1 | 1 | 1 | 1 | 0 | 1 | 1 | Low |
| Chawla 2017 | 1 | 1 | 1 | 1 | 1 | 1 | U | 1 | 1 | Low |
| Chen 2001 | 0 | 1 | 1 | 1 | 1 | 0 | 0 | 1 | 1 | Low |
| Chen 2009 | 3 | 1 | 1 | 1 | 1 | 0 | 0 | 1 | 1 | Low |
| Chow 2016 | 0 | 1 | 1 | 1 | 1 | 1 | 1 | 1 | 1 | Low |
| Crofts 2025 | 2 | 1 | 1 | 1 | 1 | 1 | 0 | 1 | 1 | Low/Moderate |
| Daniels 2016 | 2 | 1 | 1 | 1 | 1 | 0 | 0 | 1 | 1 | Low/Moderate |
| Darcy 2008 | 2 | 1 | 1 | 1 | 1 | 1 | 0 | 1 | 1 | Low |
| Das 2023 | 4 | 1 | 1 | 1 | U | 1 | 0 | U | 1 | High |
| Devaramani 2025 | 3 | 1 | 1 | 1 | U | 1 | 1 | U | 1 | Moderate |
| Disher 2017 | 0 | 1 | 1 | 1 | 1 | 1 | 0 | 1 | 1 | Low |
| Domanico 2011 | 2 | 1 | 1 | 1 | 1 | 0 | 0 | 1 | 1 | Low |
| D'Souza 2017 | 0 | 1 | 1 | 1 | 1 | U | U | 1 | 1 | Low |
| Duran 2013 | 1 | 1 | 0 | 1 | 1 | 0 | 0 | 1 | 1 | Moderate |
| Elser 2012 | 2 | 1 | 1 | 1 | 1 | 1 | 1 | 1 | 0 | Low |
| Faal 2020 | 2 | 1 | 0 | 1 | 1 | U | U | 1 | 1 | Low/Moderate |
| Freudenthal 2013 | 0 | 1 | 0 | U | 1 | 1 | 0 | U | 0 | Moderate |
| Fusch 2024 | 1 | 1 | 1 | 1 | U | 1 | 1 | U | 1 | Moderate |
| Galindo 2016 | 3 | 1 | 0 | 1 | 1 | 1 | 1 | 1 | 1 | Low |
| Garinis 2017 | 3 | 1 | 1 | 1 | 1 | 1 | 1 | 1 | 1 | Low |
| Gennattasio 2024 | 2 | 1 | 1 | 1 | U | 1 | 1 | U | 1 | Moderate |
| Gomes 2019 | 0 | 1 | 0 | 0 | 1 | 0 | 0 | U | 1 | Moderate/High |
| Hassanein 2013 | 3 | 1 | 1 | 1 | 1 | 0 | 0 | 1 | 1 | Low |
| Hernandez-Salazar 2020 | 1 | 1 | 1 | 1 | 1 | 1 | U | 1 | 1 | Low |
| Hull 2023 | 2 | 1 | 1 | 1 | U | 1 | 1 | U | 1 | Moderate |
| Ismail 2023 | 4 | 1 | 1 | 1 | U | 1 | 0 | U | 1 | Moderate/High |
| Jaguey-Hernandez 2025 | 1 | 1 | 1 | 1 | U | 1 | 0 | U | 1 | Moderate/High |
| Jayamanne 2019 | 0 | 0 | 1 | 1 | 0 | 0 | 0 | U | 1 | Moderate |
| Johnson 2003 | 0 | 1 | 1 | 1 | 1 | 1 | 1 | 1 | 1 | Low |
| Jordao 2016 | 0 | 1 | 0 | 1 | 1 | 0 | 0 | 1 | U | Moderate/High |
| Kakehashi 2007 | 4 | 1 | 1 | 1 | 1 | 1 | 0 | 1 | U | Low/Moderate |
| Kannappan 2024 | 3 | 1 | 1 | 1 | U | 1 | 0 | U | 1 | Moderate/high |
| Kargar 2017 | 4 | 1 | 0 | 1 | U | U | U | 1 | 1 | Moderate |
| Khademi 2012 | 1 | 1 | 0 | 1 | 1 | 0 | 0 | 1 | 0 | Moderate |
| Kohwaja 2022 | 2 | 1 | 1 | 1 | U | 1 | 0 | U | 1 | Moderate/High |
| Köse 2024 | 3 | 1 | 1 | 1 | U | 1 | 1 | U | 1 | Moderate/High |
| Kramaric 2017 | 4 | 1 | 0 | 1 | 1 | 1 | 0 | 1 | 1 | Low |
| Krueger 2005 | 4 | 1 | 0 | 1 | 1 | 1 | 1 | 1 | 1 | Low |
| Krueger 2007 | 0 | 1 | 1 | 1 | 1 | 1 | 1 | 1 | 1 | Low |
| Lahav 2015 | 2 | 1 | 1 | 1 | 1 | 0 | 0 | 1 | 1 | Low |
| Lam 2025 | 1 | 1 | 1 | 1 | U | 1 | 0 | U | 1 | Moderate/High |
| Lasky 2009 | 1 | 1 | 1 | 1 | 1 | 1 | 1 | 1 | 1 | Low |
| Laubach 2014 | 3 | 0 | 1 | 1 | U | 1 | U | 1 | 0 | Moderate |
| Liszka 2019 | 2 | 1 | 1 | U | U | 1 | 1 | U | 1 | Moderate |
| Liu 2010 | 2 | 1 | 1 | 1 | 1 | 1 | 1 | 1 | 1 | Low |
| Liu 2012 | 2 | 1 | 1 | 1 | 1 | 1 | 1 | 1 | 1 | Low |
| Livera 2008 | 2 | 0 | 1 | U | U | 1 | 0 | U | 1 | Moderate/High |
| Lokwani 2023 | 3 | 1 | 1 | 1 | U | 1 | 0 | U | 1 | Moderate/High |
| Lokwani 2024 | 3 | 1 | 1 | 1 | U | 1 | 0 | U | 1 | Moderate/High |
| Matook 2010 | 2 | 1 | 1 | 1 | 1 | 1 | 1 | 1 | 1 | Low |
| Mayhew 2022 | 2 | 1 | 1 | 1 | U | 1 | 0 | U | 1 | Moderate/High |
| McCallig 2024 | 2 | 1 | 1 | 1 | U | 1 | 0 | U | 1 | Moderate/High |
| Milette 2010 | 2 | 1 | 1 | 1 | 1 | 0 | 0 | 1 | 1 | Low |
| Monsen 2005 | 2 | 1 | 1 | 1 | 1 | 1 | 1 | 1 | 1 | Low |
| Mukhaiber 2025 | 1 | 1 | 1 | 1 | U | 1 | 0 | U | 1 | Moderate/High |
| Nathan 2008 | 4 | 0 | 0 | 0 | U | U | U | U | 0 | High |
| Neille 2014 | 4 | 0 | 1 | 1 | U | 1 | 0 | U | 1 | Moderate |
| Orsi 2017 | 4 | 1 | 0 | 1 | 1 | 0 | 0 | 1 | 1 | Low/Moderate |
| Pacheco 2023 | 3 | 1 | 1 | 1 | U | 1 | 0 | U | 1 | Moderate/High |
| Parra 2017 | 1 | 1 | 1 | 1 | 1 | 1 | U | 1 | 1 | Low |
| Peixoto 2011a | 4 | 1 | 1 | 1 | 1 | 1 | 1 | 1 | 1 | Low |
| Peixoto 2011b | 4 | 1 | 1 | 1 | 1 | 1 | 1 | 1 | 1 | Low |
| Philbin 2002 | 2 | 1 | 1 | U | U | 1 | 1 | U | 0 | Moderate |
| Pineda 2017 | 1 | U | 1 | 1 | U | 1 | 0 | U | 1 | Moderate |
| Pinheiro 2011 | 3 | 1 | 1 | 1 | 1 | 1 | 1 | 1 | 1 | Low |
| Pugliesi 2018 | 1 | U | 1 | U | U | 1 | 1 | U | 1 | Moderate |
| Ramesh 2009 | 4 | U | 1 | 1 | U | 1 | 1 | 1 | 1 | Moderate |
| Ramesh 2013 | 3 | U | 1 | U | U | 1 | 1 | U | 1 | Moderate |
| Ramm 2017 | 3 | U | 1 | 1 | U | 1 | 0 | U | 1 | Moderate |
| Rangaswamy 2024 | 4 | 1 | 1 | 1 | U | 1 | 0 | U | 1 | Moderate/High |
| Restin 2021 | 2 | 1 | 1 | 1 | U | 1 | 0 | U | 1 | Moderate |
| Roberts 2014 | 1 | 1 | 1 | 1 | 1 | U | 0 | 1 | 1 | Low/Moderate |
| Rodriguez-Montano 2023 | 1 | 1 | 1 | 1 | 1 | 1 | U | 1 | 1 | Low |
| Rodriguez-Montano 2024 | 2 | 1 | 1 | U | U | 1 | 1 | U | 1 | Moderate/HIgh |
| Romeu 2016 | 1 | 1 | 1 | 1 | 1 | 1 | U | 1 | 1 | Low |
| Rossi 2025 | 2 | 1 | 1 | 1 | U | 1 | 1 | U | 1 | Moderate |
| Santos 2018 | 4 | 1 | 1 | 1 | U | 1 | 0 | U | 1 | Moderate/High |
| Shariatzadeh 2021 | 4 | 1 | U | 1 | U | 1 | 1 | U | 1 | Moderate/High |
| Shoemark 2016 | 3 | 1 | 1 | 1 | 1 | 1 | U | 1 | 1 | Low |
| Slevin 2000 | 2 | 1 | 1 | 1 | U | 1 | 1 | U | 1 | Moderate |
| Smith 2018 | 4 | 1 | 1 | 1 | U | 1 | 0 | 1 | 1 | Moderate |
| Stevens 2007 | 1 | 1 | 1 | 1 | 1 | 1 | U | 1 | 1 | Low |
| Stevens 2012 | 4 | 1 | 1 | 1 | U | 1 | 1 | U | 1 | Moderate |
| Sugiura 2025 | 3 | 0 | 1 | 0 | NA | 1 | 0 | 0 | 1 | High |
| Thomas 2007 | 3 | 1 | 1 | 1 | 1 | 1 | U | 1 | 1 | Low |
| Trickey 2012 | 3 | 1 | 1 | 1 | U | 1 | 1 | U | 1 | Moderate |
| Tsunemi 2012 | 3 | 1 | 1 | 1 | U | 1 | 0 | U | 1 | Moderate/High |
| Van Enk 2011 | 3 | 1 | 1 | 1 | U | 1 | 0 | U | 1 | Moderate/High |
| Williams 2007 | 2 | 1 | 1 | 1 | 1 | 1 | U | 1 | 1 | Low |
| Williams 2009 | 3 | 1 | 1 | 1 | 1 | 1 | U | 1 | 1 | Low |
| Zamberlan-Amorim 2012 | 3 | 1 | 1 | 1 | U | 1 | 1 | U | 1 | Moderate |

**Figure s1: Representation of distribution of Risk of Bias**
